# Study protocol for “Healthy Hearts Northwest”: a 2 × 2 randomized factorial trial to build quality improvement capacity in primary care

**DOI:** 10.1186/s13012-016-0502-7

**Published:** 2016-10-13

**Authors:** Michael L. Parchman, Lyle J. Fagnan, David A. Dorr, Peggy Evans, Andrea J. Cook, Robert B. Penfold, Clarissa Hsu, Allen Cheadle, Laura-Mae Baldwin, Leah Tuzzio

**Affiliations:** 1 Group Health Research Institute, 1730 Minor Ave, Ste 1600, Seattle, WA 98101 USA; 2 Oregon Rural Practice Research Network, Oregon Health Sciences University, Portland, USA; 3 Department of Medicine, Oregon Health Sciences University, Portland, USA; 4 Qualis Health, Seattle, Washington USA; 5 Department of Family Medicine and the Institute of Translational Health Sciences, University of Washington, Seattle, USA

**Keywords:** Primary health care, Quality improvement, Cardiovascular diseases

## Abstract

**Background:**

Little attention has been paid to quality improvement (QI) capacity within smaller primary care practices which comprise nearly half of all primary care settings. Strategies for external support to build such capacity include practice facilitation (PF), shared learning opportunities, and educational outreach. Although PF has proven effectiveness, little is known about the comparative effectiveness of combining these strategies. Here, we describe the protocol of the “Healthy Hearts Northwest” (H2N) study, a randomized trial designed to address these questions while improving risk factors for cardiovascular disease.

**Methods/design:**

The targeted enrollment is 250 smaller primary care practices across Washington, Oregon, and Idaho. The study is utilizing a two-by-two factorial design to assess four different combinations of practice support: PF alone, PF with educational outreach, PF with shared learning opportunities, or PF with both. A mixed methods approach is being used for evaluation and will include data from (1) baseline and follow-up practice and staff surveys; (2) baseline and quarterly clinical performance measurement from each practice on four cardiovascular risk factors: appropriate aspirin use, blood pressure control, lipid management and smoking cessation support; and (3) a quality improvement capacity assessment (QICA) survey used by external practice facilitators to guide improvement efforts.

**Discussion:**

Results from this study will inform future large-scale practice improvement initiatives by providing comparisons of promising external practice support strategies and advance our understanding of how to build QI capacity in primary care.

**Trial registration:**

ClinicalTrials.gov, NCT02839382

## Background

Cardiovascular disease (CVD) is a leading cause of avoidable morbidity and mortality in the Unites States of America (USA) [1]. The US Department of Health and Human Services launched the “Million Hearts” initiative in 2011 to prevent 1 million heart attacks and strokes by 2017 [2]. Within the primary care setting, the initiative focuses improvement on four CVD risk factors, the “ABCS”—aspirin use in high risk patients, blood pressure control, cholesterol management, and smoking cessation counseling. In 2015, the Agency for Healthcare Research and Quality (AHRQ) launched the EvidenceNOW initiative to both support improvement of these CVD risk factors among patients within smaller primary care practices across the USA and to advance the science of building quality improvement (QI) capacity within primary care [3].

Although primary care is the foundation of health care delivery in the USA, little attention has been paid to the QI capacity of smaller primary care practices which comprise nearly half of all primary care settings [4]. These smaller practices often lack the staffing and resources to invest in the infrastructure and training that provide essential elements of QI capacity [5, 6]. Even when practices have resources and are committed to QI in principle, they often struggle with developing and implementing concrete strategies for making improvement [7]. The potential impact of building QI capacity is illustrated by the finding that twice as many deaths could be prevented by optimized ABCS risk factor control within primary care compared to optimizing acute cardiovascular care in the hospital setting [8].

Three specific external practice support strategies stand out as promising approaches to build QI capacity in primary care: practice facilitation (PF), shared learning opportunities, and educational outreach [7]. PF is delivered by a facilitator, usually external to the practice setting, who enables those who work within a practice to implement a change in care delivery [9]. It is a guided interactional process that has great potential and demonstrated ability to support uptake and application of scientific knowledge to improve clinical and managerial decision-making [10]. There is substantial evidence for the effectiveness of PF to support improvement in primary care settings [11, 12]. Shared learning opportunities, such as the learning collaborative approach pioneered by the Institute for Healthcare Improvement, can motivate change [13]. Educational outreach, often called academic detailing, involves a trained outside expert delivering one or more educational messages to a healthcare professional or the clinical team. It is generally considered a promising method of modifying health professional behavior, with a 5.6 % average improvement in guideline concordant behavior from one large systematic review [14].

Although PF alone has proven effectiveness to support improvement, little is known about the benefit of supplementing this strategy with shared learning, educational outreach, or both. This paper outlines the protocol of the “Healthy Hearts Northwest” (H2N) study, a randomized trial to build QI capacity in smaller primary care practices. The primary aim of the study is to compare the effectiveness of adding shared learning opportunities, educational outreach, or both to PF for building QI capacity within smaller primary care practices, with a focus on CVD risk factor control. Our primary hypothesis is that the improvement in the ABCS clinical performance measures will be greater among practices assigned to enhanced practice support arms of the study compared to practice facilitation alone, that practice capacity for QI will mediate this relationship, and that external organizational support and external climate for QI will moderate the observed relationship between intervention arm and change in ABCS outcomes. Our secondary hypothesis is that compared to national control practices not participating in the study, CVD clinical performance measures will improve across all practices enrolled in H2N and this improvement will vary across the different combinations of practice support.

## Methods

### Study setting and recruitment

The study is taking place from May of 2015 to April of 2018 in smaller primary care practices across three states: Washington, Oregon, and Idaho. H2N is a collaborative partnership between the MacColl Center for Healthcare Innovation at the Group Health Research Institute, Qualis Health in Washington and Idaho, the Oregon Rural Practice Research Network (ORPRN) based at the Oregon Health Sciences University, and the University of Washington’s Institute of Translational Health Sciences. Qualis Health is recruiting practices and providing PF support in Washington and Idaho. ORPRN is recruiting practices and providing PF support in Oregon. The recruitment goal is 250 practices: 120 in Washington, 100 in Oregon, and 30 in Idaho. To be eligible, practices must have 10 full-time or fewer providers in a single location and meet stage 1 electronic health record (EHR) meaningful use criteria. Our rationale for these priority criteria is to focus efforts on smaller practices with the greatest need for externally provided QI expertise and have some capability to produce clinical performance measure reports at the start of the study, given the relatively short 36 months of funding.

### Study design

**Fig. 1:**
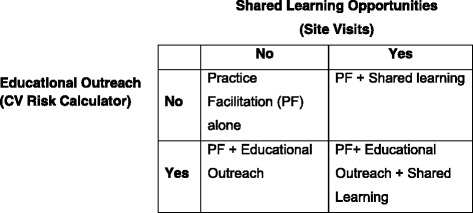
Factorial study design

A two-by-two factorial design is being used to compare the effectiveness of adding shared learning opportunities and educational outreach to PF. (Fig. 1) The four factors (intervention arms) are (1) PF alone, (2) PF and shared learning, (3) PF and educational outreach, and (4) PF combined with both shared learning and educational outreach. In addition, a set of control data for the ABCS clinical performance measures will be obtained from a randomly selected national sample of primary care practices not participating in the study. This control data will consist of the four ABCS clinical performance measures over the same historical time period and reporting intervals as the practices enrolled in the H2N study. Control practices will be matched on practice size (clinician FTE), QI measure reporting capabilities, and rural/urban location. This data will be provided by the DARTNet Institute [15].

### Practice support interventions

PF support is provided to all sites upon enrollment in the study for 15 months as a unifying approach. Two enhanced practice support interventions, shared learning through site visits and educational outreach on CVD risk calculator use, are being offered to those practices randomized to receive them over a period of 4 months approximately 3–9 months after the start of the PF support. A description of each type of external practice support follows.

### Practice facilitation and support

A geographic cluster of 10–20 practices are assigned to each practice facilitator. Each practice is receiving 15 months of support with a minimum of five face-to-face visits from the facilitator, with a monthly phone call in-between the face-to-face visits. Additional phone or video calls, emails, and text messaging are being provided as needed. A practice self-assessment tool (see measures below) is used by the facilitator in the first face-to-face visit to help the practice team achieve consensus on their current status for each of eight change concepts that build on prior practice transformation work in small primary care practices [16]. These change concepts provide direction to small practices in their efforts to transform into true learning and improvement organizations and comprise activities in the eight areas as described in Table 1. More than a decade of work by members of our team supports these concepts as foundational to build practice capacity to learn and improve [17, 18].

**Table 1:** Quality improvement change concepts and key activities

Change concept	Description of practice activities
Embed clinical evidence on ABCS into daily work to guide care for patients	▪ Review the evidence supporting the ABCS for primary and secondary prevention of cardiovascular risk ▪ Review treatment guidelines for ABCS measures ▪ Educate staff on clinical guidelines ▪ Select patient education materials for primary and secondary prevention
Utilize reliable, robust data to understand and improve ABCS measures	▪ Develop process to pull data from EMR ▪ Review data for accuracy and build confidence in data ▪ Develop process to support accurate data entry/collection ▪ Use data to identify gaps between the evidence-based guidelines and current care for all patients on panel ▪ Create population-based reports and visual data dashboards
Establish a regular QI process involving cross-functional teams	▪ Set aside regular meeting time for cross-functional QI team ▪ Select a QI methodology to structure improvement efforts ▪ Train team members on QI methodology ▪ Practice good meeting skills ▪ Regularly review data on ABCS outcome and process measures to understand areas for improvement ▪ Invite patient(s) to participate on the QI team
Identify at-risk patients for prevention outreach	▪ Understand current patient panel relative to ABCS ▪ Select actionable improvement goals based on ABCS data ▪ Recall patients overdue for care/outreach related to ABCS testing, education, counseling
Define roles and responsibilities (tasks) across the care team to identify and manage ABCS population	▪ Use workflow mapping to examine current processes and explore other approaches ▪ Introduce preventive screenings and educational materials for ABCS measures into workflow ▪ Develop/enable point of care reminders based on ABCS guidelines ▪ Scrub charts daily to flag patients needing support on ABCS
Deepen patient self-management support for action planning around ABCS	▪ Train staff in motivational interviewing ▪ Develop shared care plans with patients, emphasizing goal setting led by patient values ▪ Follow up with patient progress toward care plan goals
Develop robust linkage to smoking cessation, self-management programs, and other evidence-based community resources	▪ Create list of community resources and keep in a location accessible to all staff members ▪ Outreach to community resources to build referral pathway ▪ Provide list of resources to patients ▪ Proactively refer patients to community resources and assist in establishing patient with the resource

*ABCS* aspirin, blood pressure, cholesterol, smoking, *EMR* electronic medical record, *QI* quality improvement

### Shared learning opportunities through site visits

Practices randomized to the shared learning intervention arm are offered the opportunity to visit an exemplar practice with a particularly strong or innovative approach to QI. Exemplars are identified through nominations from facilitators and other members of the H2N collaborative. They may or may not be practices enrolled in H2N but must be within the three state geographic region of the study. Exemplars are screened by phone about their QI program and asked for reports on their clinical performance measures. Practices randomized to visit an exemplar receive support to coordinate the site visit and travel reimbursement. Each exemplar practice hosts 4–6 practices for a half-day visit within the 4-month intervention period. The goal of the site visit is to create an opportunity for practices to directly observe their QI approach and tools, examine work flows and team member roles, and develop an on-going relationship with a practice that has an innovative approach to QI.

### Educational outreach

The purpose of the academic educational outreach is to encourage use of a CVD risk calculator or estimator [19] within patient encounters. Current evidence-based clinical guidelines support the use of such a risk estimator to inform decisions about use of a statin medication to manage CVD risk based on age, gender, smoking and diabetes status, lipoprotein levels, and blood pressure [20]. A small advisory group of full-time primary care clinicians are developing the content of the educational outreach program to address priority topics and issues. The educational outreach will begin with a brief introductory video example of how a CVD risk estimator can be integrated into a patient visit. This will be followed by a 30-min phone call between each clinical team in practices randomized to the intervention and a physician academic expert. Each academic expert will be assigned 15 to 20 practices for a phone call.

### Data collection and measures

Sources of data, measures, and variables are listed in Table 2. A single practice-level survey and a survey completed by all clinicians and practice staff are administered at baseline, 4 months after the period of the enhanced practice support and 6 months after withdrawal of all practice support activities. The practice survey includes practice demographics, information about health IT systems, and the Change Process Capacity Questionnaire (CPCQ), a measure of the ability of an organization to manage change processes [21]. The staff member survey includes the adaptive reserve scale, a measure of internal capability for organizational learning and development [22].

**Table 2:** Measures and data sources

Construct	Data source	Measure(s)	Timing
Practice capacity for quality improvement (QI)	Quality improvement capacity assessment (QICA) survey	• Eight change concepts (see Table 1)	Baseline and 9–12 months after start of practice facilitation
Prior experience with QI	Practice survey	• Change process capacity questionnaire (CPCQ) [21]	Baseline and 4 months after exposure to enhanced support interventions
External organizational support for QI	Practice survey	• Is the practice is part of a large organization with a centralized QI team? • The autonomy of the practice to choose what QI projects they wish to work on	Baseline and 4 months after exposure to enhanced support interventions
External climate for QI	Practice survey	• Location of practice: Washington, Oregon, or Idaho	Baseline and 4 months after exposure to enhanced support interventions
Adaptive reserve	Staff survey	• Adaptive reserve scale [22]	Baseline and 4 months after exposure to enhanced support interventions
Clinical quality measures for ABCS CVD risk factors	Numerator and denominator report generated by each practice from their Electronic Health Record	• NQF0068: ischemic vascular disease: appropriate use of aspirin/antithrombotic • NQF0018: controlling high blood pressure • NQF0028: preventive care and screening: tobacco use • CMS proposed statin measure	Every 90 days with a 12 month look-back period

*CVD* cardiovascular disease; *NQF* National Quality Forum; *QI* quality improvement; *ABCS* aspirin, blood pressure, cholesterol, and smoking

CVD clinical quality measures (CQMs) on each of the ABCS risk factors are being submitted by each practice every 3 months with a 12-month look-back period for each submission. Each practice submits a numerator and denominator for each CQM every 90 days. Three of the CQMs are based on definitions provided by the National Quality Forum (NQF): NQF0068: ischemic vascular disease: appropriate use of aspirin/antithrombotic [23]; NQF0018: controlling high blood pressure [24]; and NQF0028: preventive care and screening: tobacco use: screening [25]. The original NQF cholesterol measure proposed for the study is under revision based on recent changes in evidence-based clinical guidelines. Instead, we are requesting data from each practice on the proportion of patients prescribed a statin who (a) have diabetes; (b) have a history of ischemic vascular disease; or (c) in the absence of either condition have a low-density lipoprotein (LDL) value greater than 190 mg/dl [26].

Practice capacity for QI activities are measured at two time points by the quality improvement capacity assessment (QICA) tool, a practice self-assessment tool used by the facilitators to help practices identify areas where change is needed to improve their capacity to engage in QI. It is a modification of a previously validated tool used to guide primary care practice transformation into a medical home [16]. The single QICA for each practice is completed by the practice team as a group during the first face-to-face visit with their facilitator and again at the fourth of the five face-to-face quarterly visits.

External organizational support for QI is reflected in the growing influence of individual practice relationships with other practices, hospitals, and healthcare systems. Two questions on the practice survey are used to assess this external organizational support: the degree to which the practice is part of a larger organization with a centralized QI team and the autonomy of the practice to choose what QI projects they wish to work on.

External climate for QI is indicated by the state within which the practice is located. Washington, Oregon, and Idaho all provide unique state contexts and history with primary care support, transformation, innovation, and medical home work. For example, Oregon is currently a site for the CMS/CMMI Comprehensive Primary Care Initiative. Washington and Idaho both received a State Innovation Model grant in 2014 from CMS. Idaho is supporting primary care medical homes as part of their SIM grant, but not Washington.

Qualitative data: We are using observation, interviews, and field notes to describe how delivery of QI support through PF was tailored to individual practices, the influence of the external climate, as well as challenges, success factors, and lessons learned. Practice facilitators are keeping detailed notes after each contact with their assigned practices including face-to-face visits, phone calls, emails, and text messages. In addition to these field notes, additional data are being collected through focus groups with facilitators at training sessions, interviews with experts in state level healthcare policy, and interviews with individual facilitators.

### Randomization and data analysis

#### Randomization

We are using a stratified randomization with enrolled practices categorized into one of six strata defined by PF support organization (Qualis Health or ORPRN), prior practice experience obtaining customized data to drive improvement (yes, no), and prioritization of the work by the practice (high, low). Information on the latter two variables is collected in the baseline practice survey prior to randomization. Within each stratum, practices are randomly assigned by a computer-generated randomization scheme to one of the four intervention arms developed by the biostatistician. The evaluation/analysis team [AJC, RP, AC] are blinded to assignment.

#### Data analysis

**Fig. 2:**
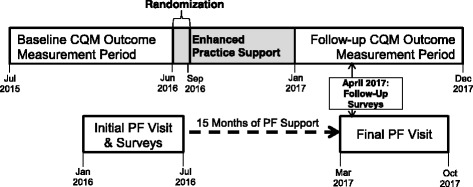
Project timeline and data collection windows

The primary aim of this study is to compare the effectiveness of adding shared learning opportunities, educational outreach, or both to PF on CQMs for each ABCS risk factor by building QI capacity within each practice. To address this aim, our primary outcome is the practice-level blood pressure CQM covering the time period 1-year after intervention uptake. Specifically, all practices participating in the study are randomized to one of four intervention groups in August 2016. We define baseline ABCS CQMs as those covering the year prior to randomization (July 1, 2015 to June 30, 2016) (Fig. 2). After randomization, roll-out of the active intervention arms occurs from September 2016 to December 2016. CQM data that covers this roll-out time period for all intervention arms will not be used for outcome assessment to allow for active intervention uptake (similar to a wash-out period in a standard cross-over randomized trial). Our 1-year follow-up outcome measure for our primary aim is the blood pressure CQM that covers the 12-month time period after the intervention roll-out period, from January 2017 to December 2017.

For the primary analysis to assess differences between the four intervention groups on the primary outcome practice-level proportion with blood pressure control at 1-year follow-up (blood pressure CQM), we will fit the following linear regression model:

$$ {Y}_i={\beta}_0+{\beta}_1{X}_{1i}+{\beta}_2{X}_{2i}+{\beta}_3{X}_{3i}+{\beta}_{\mathrm{base}}{Y}_i^0+{\boldsymbol{\beta}}_z{\boldsymbol{Z}}_{\boldsymbol{i}}+{e}_i\ {e}_i\sim N\left(0,{\sigma}^2\right)\ i=1,\dots, N $$

where *Y*_*i*_ is clinics *i* blood pressure CQM at 1-year follow-up, *X*_1*i*_, *X*_2*i*_, and, *X*_3*i*_ are three indicator variables for intervention assignment for site visit, educational outreach, or both, respectively, *Y*_*i*_^0^ is baseline blood pressure CQM, and ***Z***_***i***_ is a vector of other baseline covariates to adjust for differences between clinics including PF support organization (Qualis Health or ORPRN), baseline prior practice experience obtaining customized data to drive improvement (yes, no), and baseline prioritization of the work by the practice (high, low). We will use the Fisher’s protected least significant difference approach to control for multiple comparisons due to the comparison of four intervention arms. Specifically, we will first calculate an Omnibus *F* test to assess if there are any significant differences between intervention groups and only calculate pairwise comparisons if this omnibus test is statistically significant.

Our analyses will assume intention to treat principles by treating intervention assignment as randomized regardless of whether the intervention had uptake within the practice. We will also attempt to obtain outcome data for all practices even if they stop participating in the intervention. However, there may be some practices that drop-out and stop providing follow-up data. To account for bias due to loss-of-follow-up, we will adjust for baseline practice-level variables that we a priori expect to be related to outcome and may be predictive of loss-to-follow-up. Sensitivity analysis including last value carry forward (follow-up outcome is the baseline outcome for missing practice data) will also be conducted. If loss-to-follow-up is large (>15 %), we will further explore inverse probability of treatment weighting or multiple imputation to handle missing outcome data. We will further conduct subgroup analyses to assess for differential intervention effects (moderators) by baseline practice-level variables including PF support organization, prior practice experience obtaining customized data to drive improvement, prioritization of the work by the practice (high, low), and adaptive reserve (low, medium, and high). Similar analysis will be conducted for secondary outcomes including the other ABCS CQM measures and changes in practice variables such as practice capacity QI.

If we find a difference between any of the four intervention arms, we will conduct a mediator analysis to assess if change in practice capacity for QI was a potential pathway for change in ABCS CQM outcomes. Specifically, we will conduct a mediator analysis following the Barron and Kenny framework [27]. Step 1 is to show that the intervention had an effect of the follow-up outcome. Step 2 is to show that the intervention was associated with change in the potential mediator practice capacity for QI. Step 3 is then to show that the intervention effect is mediated or reduced once the mediator is taken into account. For step 2, we will use a similar linear regression model as outlined for the primary analyses, but the outcome will be the mediator change in practice capacity for QI. For step 3, we will use a similar linear model as outlined for the primary analysis except now further adjust for the change in practice capacity for QI. We will use the method of Sobel [28] to test for the mediator’s indirect effect of the interventions on the ABCS CQM outcome.

A secondary aim of this study is to assess if PF alone improves clinic-level ABCS outcomes (note that PF is given to all practices in our randomized trial population). To address this aim, we will conduct an observational study using data from our randomized study practices and control practices from the DARTNet Institute. Specifically, we will have data on practice-level ABCS outcomes 1-year prior to PF roll-out in each of our randomized trial practices and 1-year post PF roll-out. To control for potential confounding, we will match each randomized control trial practice to one or more control practices matching on practice size (clinician FTE), QI measure reporting capabilities as measures by their ability to generate a custom report without support from their EHR vendor, and rural/urban location. For each matched pair, we will use the same data ascertainment windows based on when the randomized control trial practice had PF rolled out. This will control for temporal changes. We will conduct similar analyses as proposed for the randomized trial except that now outcome time is tied to PF roll-out, and we will only include a single intervention indicator that had or did not have PF.

### Sample size

The main aim of the trial is to assess if any of the enhanced practice support arms (site visit, educational outreach, or both) improved ABCS CQM outcome performance relative to practices that received PF alone. If one assumes a 20 % attrition rate over the life of the study leaving 50 practices in each of the four intervention arms, we have 80 % power to detect a 0.114 proportion improvement in any of the three enhanced practice support arms relative to PF for the 1-year practice outcomes blood pressure CQM or aspirin CQM. We assumed an outcome standard deviation of 0.26 for both ABCS outcomes (based on baseline data from the enrolled practices) and a *F* test comparing the four intervention arms assessing for any difference between groups. Further, we used an adjusted sample size calculation for ANCOVA models [29] (proposed analysis adjusts for baseline outcome) assuming a 0.60 correlation coefficient between baseline outcome measure and follow-up outcome measure. Even with an additional 10 % clinic attrition rate (45 practices per arm with follow-up), we will have 80 % power to detect a difference of 0.121 in practice proportion improved in either ABCS outcome. We conducted power calculations via simulation using R version 3.0.2.

### Trial status

At the time of manuscript submission, the study was 16 months into the 36 months of support from AHRQ. The Group Health Research Institute’s Institutional Review Board approved this study.

## Discussion

### Significance

While prior research identified promising approaches for supporting primary care practice improvement in the USA, there have been few comparative effectiveness studies that rigorously assessed the combinations of the external practice support interventions described here. In a study that compared traditional learning collaborative with PF to PF alone, the addition of a local learning collaborative enhanced use of asthma guidelines [30]. In addition, little is known about effective and efficient approaches to support practice improvement across a large geographic region with the number and diversity of smaller primary care practices enrolled in Healthy Hearts Northwest [31, 32].

Evaluations of the effectiveness of the traditional “learning collaborative” approach to shared learning have shown mixed results [33, 34]. Our unique approach to shared learning through site visits is grounded in diffusion of innovation theory on the premise that such visits create opportunity to more directly observe QI approaches and understand how these can be adapted to a practice’s own circumstances and setting [35]. Site visits may also serve to alter informal peer social networks by establishing or strengthening ties between individuals across practices or clinics [36]. In one of the few studies of the influence of a social network, Keating and colleagues demonstrated that physicians were more likely to obtain information from colleagues with greater expertise and experience [37].

The role of educational outreach in addition to practice facilitation and or shared learning activities is largely unknown. Traditional educational outreach is a one-on-one activity between an academic expert and an individual clinician. In a study of group versus individual academic detailing for use of antihypertensive medications, both were equally more effective than usual care practices in improving prescribing habits [38]. For purposes of this study, we will be using educational outreach to help facilitate a major shift in the way that the clinicians and their teams approach all four ABCS risk factors by combining them into a single CVD risk measure. The alternative is to conduct educational outreach for each individual ABCS measure separately. If successful, this shift in approach would be a significant step forward in implementation methods.

### Limitations

In addition to recruitment and retention barriers, potential limitations to building the QI capacity include a lack of EHR resources needed to generate ABCS clinical performance measures even though the system may meet stage 1 EHR meaningful use criteria, no financial incentives for investing in building QI capacity, and the competing demands of the changing reimbursement environment such as implementation of the Medicare Access and CHIP Reauthorization Act [39]. In addition, disruptions such as implementing a new EHR, turnover in practice leadership, changes in practice staffing, or practice acquisition by a larger healthcare organization pose significant barriers to building high-functioning QI teams. We will mitigate EHR limitations by providing specific consultations and technical assistance by information technology experts from both Qualis Health and the Oregon Health Sciences University. Practice facilitators will work closely with their assigned practices to assess additional opportunities to participate in other practice transformation support initiative and assess their synergy with the Healthy Hearts Northwest support as well as maintaining trusting relationships with all members of the practice team to assist them with disruptions when they occur.

### Impact

In addition to its impact on CVD across a large population in the Pacific Northwest, the work of improving the capacity of smaller practices to engage in QI activities will prepare them for the transition to value-based reimbursement. Our findings will also have broad implications for understanding the type of technical assistance and support required to support smaller primary care practices in a rapidly changing healthcare environment.

## Abbreviations

ABCS: Aspirin, blood pressure, cholesterol, smoking
AHRQ: Agency for Healthcare Research and Quality
CPCQ: Change Process Capacity Questionnaire
CVD: Cardiovascular disease
EHR: Electronic health record
H2N: Healthy Hearts Northwest
LDL: Low-density lipoprotein
NQF: National Quality Forum
ORPRN: Oregon Rural Practice Research Network
PF: Practice facilitation
QI: Quality improvement
QICA: Quality improvement capacity assessment
USA: United States of America
